# One Step Forward in Understanding the Mechanism of Action of Wood Vinegar: Gas Exchange Analysis Reveals New Information

**DOI:** 10.3390/plants15020262

**Published:** 2026-01-15

**Authors:** Sara Desideri, Lisa Grifoni, Riccardo Fedeli, Stefano Loppi

**Affiliations:** 1 BioAgryLab, Department of Life Sciences, University of Siena, 53100 Siena, Italy; s.desideri2@student.unisi.it (S.D.); lisa.grifoni@unisi.it (L.G.); loppi@unisi.it (S.L.); 2 Department of Life Sciences, University of Siena, 53100 Siena, Italy; 3 Istituto Nazionale Geofisica e Vulcanologia (INGV), 605, Via di Vigna Murata, 00143 Rome, Italy; 4 National Biodiversity Future Center, 90133 Palermo, Italy

**Keywords:** abiotic stress, eustress, gas exchange, pyroligneous acid, wood distillate

## Abstract

Wood vinegar (**WV**), a by-product of woody biomass pyrolysis, is increasingly used in agriculture as a sustainable biostimulant, although its effects on plant stress resistance and underlying mechanisms remain poorly understood. Recent studies propose that **WV** may act through a *eustress*-based mechanism, defined as a mild and controlled stress that activates adaptive physiological responses and enhances plant performance without causing structural or metabolic damage. This study investigated the physiological and biochemical effects of **WV** on strawberry plants grown under three water-deficit stress levels [no stress (**NS**), moderate stress (**MS**), and high stress (**HS**)] and treated with **WV** either via fertigation (0.5% *v*/*v*, **WV_1_**) or foliar spray (0.2% *v*/*v*, **WV_2_**). Gas exchange parameters (**A**, **g_sw_**, **E**, **Ci**, **WUE**), total chlorophyll content, and nutrient balance ratios (**Fe/Mn** and **K/Ca**) were measured after a three-month growth period. PERMANOVA revealed significant effects of both **WV** and water-deficit stress, as well as their interaction, on most parameters. Under **NS** and **MS** conditions, **WV** reduced **A**, **gsw**, **E**, and **Ci** while increasing **WUE**, indicating enhanced water-use efficiency and improved physiological adjustment to water limitation. Chlorophyll content remained stable, demonstrating preserved photosynthetic integrity. Nutrient ratios further supported a controlled ion rebalancing associated with adaptive stress responses under **NS** and **MS**, whereas **HS** conditions indicated the onset of distress. Overall, the data demonstrate that **WV** enhances plant stress resistance primarily by inducing eustress-mediated physiological regulation rather than by directly stimulating growth.

## 1. Introduction

Wood vinegar (**WV**) is a bio-based product obtained through pyrolysis, a thermochemical process in which woody biomass is burned under limited or no oxygen presence [1,2,3]. The resulting gases are condensed in an external tank in the form of an amber liquid, rich in >300 compounds (i.e., polyphenols, organic acids, esters, ketones, etc.) [4]. The chemical characteristics of **WV** are influenced by different factors, including the biomass feedstock, heating rate, pyrolysis temperature, and the configuration of the condensation systems. Based on all these factors, different types of **WV** can vary significantly, leading to different effects when used as bio-stimulants in agriculture [5,6].

In recent years, **WV** has been used in agriculture as an environmentally friendly bio-stimulant to improve plant growth and yield [7,8,9,10] serving as a sustainable alternative to chemical fertilizers. The use of this type of products is also supported by supranational organizations, such as the European Commission (EC), which introduced several important targets to reduce the use of chemical pesticides and fertilizers [11,12]. Beyond its agronomic relevance, **WV** has gained interest as a low-input tool potentially capable of enhancing plant resilience under sub-optimal environmental conditions.

Following this wave, scientific research, according to Scopus data, has marked an upward trend since 2020, with >50 papers published in international journals linked to the keywords “*wood vinegar*”, “*pyroligneous acid*”, “*wood distillate*” [13]. Most studies have investigated the benefits of this by-product on crops directly evaluating its potential to influence the photosynthetic parameters and the metabolism of plants rather than elucidating its mechanism of action [14,15,16]. This gap is primarily related to the complex chemical composition of the **WV**, which significantly complicates the attribution of effects to individual components [17]. As a result, **WV** is increasingly interpreted as a functional mixture capable of triggering integrated physiological responses rather than acting through single-molecule effects.

In our previous research [18,19,20], we proposed *eustress* as a possible mechanism of action for **WV**. *Eustress* is a form of mild, beneficial stress that activates positive adaptive responses in plants [21,22], whereas *distress* leads to irreversible impairment of photosynthetic performance and structural integrity [23]. The *eustress* hypothesis suggests that biostimulants’ chemical composition may contain compounds that stimulate defense pathways and enhance antioxidant activity, “*priming*” the plants to better withstand drought, pathogens, or nutrient imbalances without causing damages [24,25,26].

Fedeli et al. [19] showed that plants sprayed weekly with 0.2% **WV** exhibited stable or increased chlorophyll content at the end of the experiment, but, after the first **WV** application, a reduction in the photosynthetic parameters (F_v_/F_m_ and PI_abs_) was observed. Additionally, Noel et al. [27] supported the *eustress* hypothesis, showing that **WV** increased CO_2_ fixation and enhanced the synthesis and turnover of different photosynthetic pigments, specifically the β-carotene, a key antioxidant involved in photoprotection. Moreover, it was also observed that **WV** raises both the leaf Chl*_a_*/Chl*_b_* ratio and the root nodulation in soybean (*Glycine max* (L.) Merr. subsp. max), a further indication of an adaptive adjustment typical of the *eustress* responses rather than a simple enrichment effect.

Drought stress represents one of the most relevant abiotic constraints in crop production and provides an appropriate physiological framework to test the eustress hypothesis. Water deficit severity can range from mild to severe, triggering progressive responses such as stomatal regulation, osmotic adjustment, activation of antioxidant defenses, and hormone-mediated signaling, particularly involving abscisic acid. Under moderate drought, plants often prioritize adaptive strategies that preserve photosynthetic efficiency and water-use efficiency, whereas severe drought leads to metabolic disruption and growth inhibition. Recent syntheses emphasize that drought tolerance relies on the coordinated regulation of stomatal behavior, redox homeostasis, and hormonal crosstalk rather than on single traits alone [28].

Within this context, **WV**-induced *eustress* may act as a priming stimulus, enhancing plant preparedness to cope with water deficit by pre-activating defense and regulatory pathways without imposing the metabolic costs associated with strong stress responses.

Based on this evidence, this study aimed to investigate the mechanism of action of **WV** in two different application methods (i.e., fertirrigation and foliar spray) after the post-fruiting stage (three months of growing period) of strawberry (*Fragaria* × *ananassa* (Duchesne ex Weston) Duchesne ex Rozier, cv ANIA) used as a model plant, under different water stress conditions. Gas exchange (i.e., net CO_2_ assimilation rate (**A**), stomatal conductance (**g_sw_**), transpiration rate (**E**), intercellular CO_2_ concentration (**Ci**), intrinsic water-use efficiency (**WUE**)), total chlorophyll, and nutrient balance ratios (i.e., **Fe/Mn** and **K/Ca**) were measured to understand whether **WV** elicits beneficial low-level stress responses under normal and abiotic conditions.

## 2. Results and Discussion

The application of **WV** with both application methods (i.e., **WV_1_**: fertirrigation; **WV_2_**: foliar spray) significantly impaired the physiological and biochemical patterns of strawberry plants, with similar values alone or in combination with water stress (Table 1), supporting the idea of the *eustress* mechanism of action of the **WV** [19].

### 2.1. Gas Exchange

**Figure 1:**
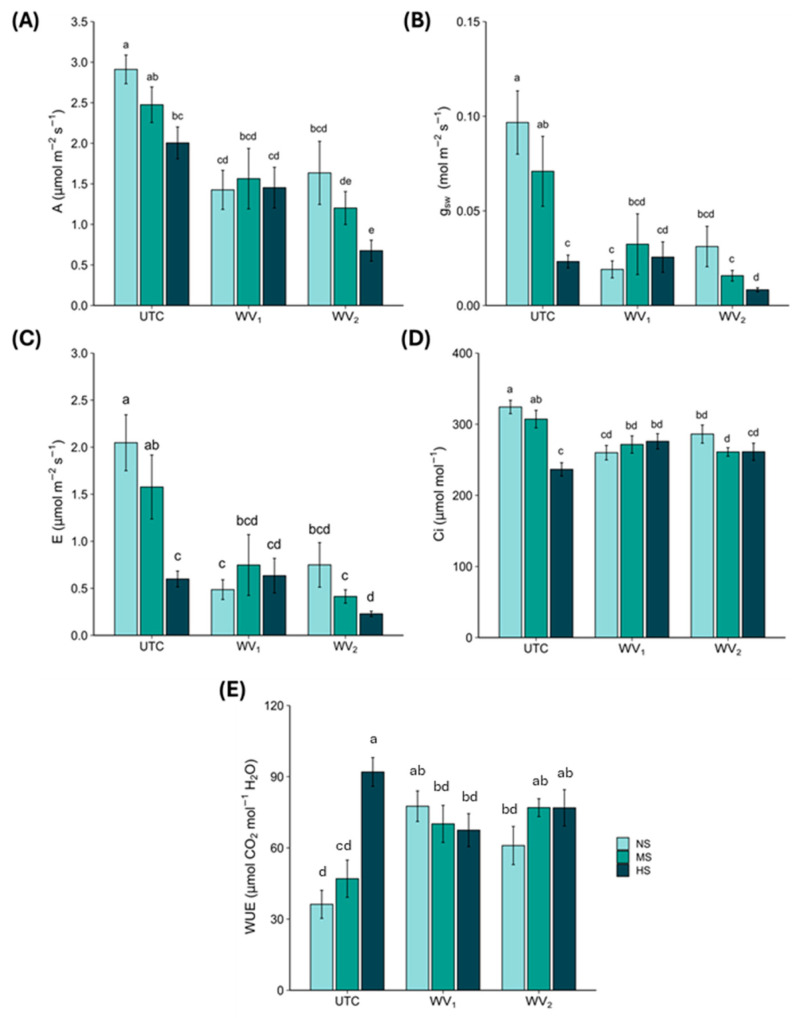
Leaf gas exchange parameters (mean ± standard error): (**A**) net CO_2_ assimilation rate (**A**), (**B**) stomatal conductance (**g_sw_**), (**C**) transpiration rate (**E**), (**D**) intercellular CO_2_ concentration (**Ci**), (**E**) intrinsic water-use efficiency (**WUE**), **UTC** = plants untreated, **WV_1_** = plants fertigated with wood vinegar at 0.5% (*v*/*v*), and **WV_2_** = plants foliar sprayed with wood vinegar at 0.2% (*v*/*v*). **NS** = no stress, **MS** = moderate stress, and **HS** = high stress. Different letters indicate statistically significant (*p* < 0.05) differences between treatments.

**Figure 2:**
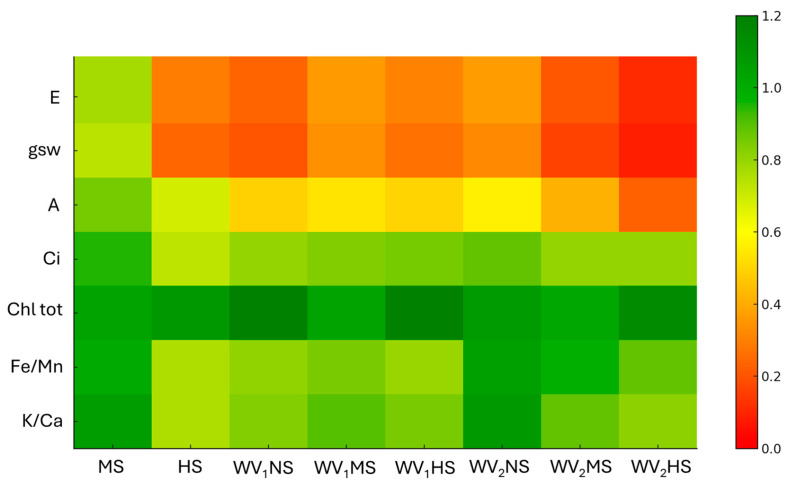
Heatmap showing the change in gas exchange parameters (**A**, **g_sw_**, **E**, **Ci**, **WUE**), chlorophyll content (**Chl tot**), and nutrient balance ratios (**Fe/Mn** and **K/Ca**) under different water stress treatments alone (**MS** and **HS**) and in combination with wood vinegar (**WV**) in both fertigation (**WV_1_**) and foliar spray application (**WV_2_**) expressed as ratio respect to the control (**NS**). Green shades indicate an increase, while red shades represent a decrease compared to **NS**. The intensity of the color reflects the magnitude of the variation.

**Figure 3:**
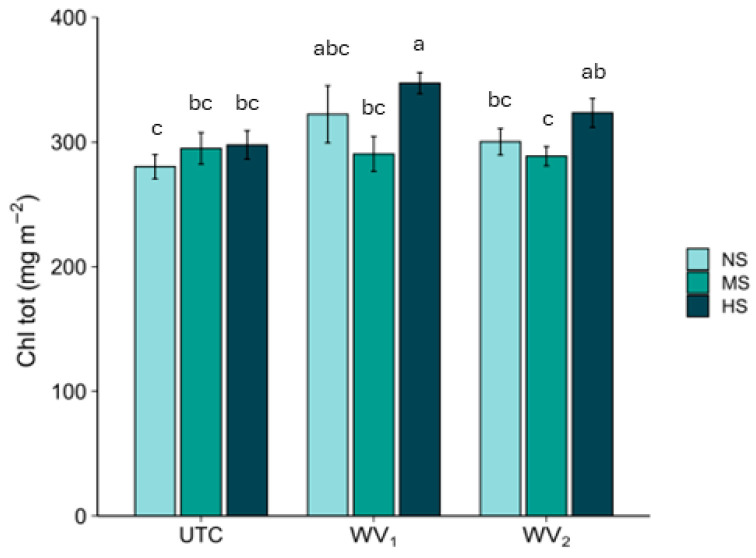
Total chlorophyll content (**Chl tot**) (mean ± standard error). **UTC** = plants untreated; **WV_1_** = plants fertigated with wood vinegar at 0.5% (*v*/*v*); and **WV_2_** = plants foliar sprayed with wood vinegar at 0.2% (*v*/*v*). **NS** = no stress, **MS** = moderate stress, and **HS** = high stress. Different letters indicate statistically significant (*p* < 0.05) differences between treatments.

At **NS** condition, on one hand both **WV** treatments showed significant reduction in **A**, **g_sw_**, **E**, and **Ci** (**WV_1_**: −51%, −80%, −76%, −20%,, respectively, and **WV_2_**: −44%, −68%, −63%, −12%, respectively) compared with **UTC** (Figure 1A–D, 2). On the other hand, a statistically significant increase was reported for **WUE** in both **WV_1_** (+114%) and **WV_2_** (+68%) compared with **UTC** (Figure 1E, 3).

At **MS** condition only **WV_2_** determined a statistically significant reduction in **A**, **g_sw_**, **E**, and **Ci** (−51%, −78%, −74%, −15%, respectively) compared with **UTC** (Figure 1A–D, 2). Regarding **WUE, WV_2_** showed a statistically significant increase (+64%) compared with **UTC** (Figure 1E, 2).

A similar pattern was observed at **HS**, in which **WV_2_** caused a statistically significant reduction in **A**, **g_sw_**, and **E** (−66%, −64%, −62%, respectively) compared with **UTC** (Figure 1A–C, 2); instead **WV_1_** showed a significant reduction in **WUE** (−27%; *p* < 0.05). Differently, both **WV_1_** (+17%; *p* < 0.05) and **WV_2_** (+11%; *p* > 0.05) showed an increase in **Ci** compared with **UTC** (Figure 1E, 2).

From a general perspective, the above results showed a clear and coherent physiological response. In both application methods, **WV** triggered a water-saving mechanism in the plants, concerning the stomatal regulation (reduction in **g_sw_** and **E**) under *eustress* conditions [29,30]. The reduction of **A** is generally attributed to photosynthetic damage [31,32]; however, in our case, the total chlorophyll content (see Section 3.2) did not vary among the **WV**-treated and **UTC** plants. The observed reduction of **A** could be attributed to a direct consequence of the minor availability of CO_2_ caused by stomata closure, as already shown by several research studies [32,33].

**Ci** showed two different responses to the tested treatments. As concerns **NS** and **MS**, we observed a decrease of **Ci** in **WV**-treated plants, in which the reduction of **g_sw_** limited CO_2_ entrance and resulted in a corresponding reduction in **A** [34,35]. In contrast, in **HS**, we observed an increase in **Ci**, suggesting the onset of non-stomatal limitations. Indeed, CO_2_ seems to be no longer used in the biochemical processes of photosynthesis, showing the beginning of a *distress* [34,35], despite, in our case, the chlorophyll content did not decrease (see Section 2.2).

The hypothesis of *eustress* was also supported by the results of **WUE**, since its increase reported in **NS** and **MS** suggests that the **WV**-treated plants were able to ameliorate the efficiency of the water use. This pattern, together with **g_sw_**, **E**, and total chlorophyll content, points out a clear and coherent response to the *eustress* hypothesis, whereby the plants reduced the gas exchange for an optimization of the water saving, without inducing impairments in the photosynthetic apparatus (see Section 2.2).

### 2.2. Chlorophyll

Chlorophyll content showed no statistically significant variation among the treatments, with the only exception in **HS**, in which **WV_1_** showed a significant increase (+8%) compared with **UTC** (Figure 2 and Figure 3).

Several studies reported an increase in chlorophyll content due to the application of **WV**, in both foliar and fertirrigation methods. For example, Hur et al. [36] and Mohd Amnan et al. [37] reported a significant increase in chlorophyll content in *Oryza sativa* L. and *Pandanus amaryllifolius* Roxb., respectively, following foliar applications of **WV**. Similarly, Abdel-Sattar et al. [38] and Afsharipour et al. [39] observed increases in *Anthracothorax mango* L. and *Cucumis sativus* L. when **WV** was applied through fertigation. The lack of variation in chlorophyll content in our research was consistent with the results reported by Fedeli et al. [19] in which the first applications of **WV** determined a reduction in the key photosynthetic parameters (i.e., F_v_/F_m_ and PI_abs_), followed by a progressive recovery. After several applications, both parameters returned to control values, indicating an absence of structural damage to the PSII [19]. Since F_v_/F_m_ and PI_abs_ are common indicators in the photosynthetic efficiency [40,41,42], these results highlight once again that **WV** impacts the plants positively, without inducing *distress*. This perspective was also coherent with previous studies showing that **WV** raises the antioxidant content in plants (leaves, roots and fruits; [43,44,45,46,47]). These effects are a clear indication of an *eustress* effect that contributes to maintaining the integrity of the PSII functionality and also reflects an enhancement in growth, yield, and nutritional parameters of the plants.

### 2.3. Nutrient Balance Ratios

Under **NS** conditions, **WV_1_** showed a significant reduction (−17%) in **K/Ca**; at **MS** conditions, both **WV** treatments showed a significant reduction in **K/Ca** (**WV_1_**: −16%; **WV_2_**: −18%); under **HS** conditions, no significant differences were observed among the treatment **K/Ca**. No statistically significant differences were reported in **Fe/Mn** ratio in any condition (Table 2, Figure 2).

The nutritional ratios investigated (i.e., **K/Ca** and **Fe/Mn**) are widely used for the evaluation of ionic balance and nutrient homeostasis in plants [48,49,50]. **Fe/Mn** is an indicator of micronutrient homeostasis, since a reduction in this ratio reflects the limited availability of **Fe** or a relative increase in **Mn** content, with negative effects on the plant’s photosynthetic systems [51,52]. Differently, **K/Ca** is an indicator of cation balance, as a decrease in this ratio reflects a prevalence of **Ca** over **K**, suggesting an enhancement of structural stability of cell walls but a reduced osmotic capacity, potentially affecting stomatal regulation and gas-exchange efficiency under stress [53,54]. Since these ratios generally reflect the ability of plants to maintain a stable ionic balance under stressful conditions, they allow the interpretation of the type of stress to which plants are subjected, *eustress* or *distress* [55,56,57]. The **Fe/Mn** showed a slight but not significant decrease in both **UTC** and **WV**-treated plants across the levels of the water stress (**NS** → **MS** → **HS**; Table 2), indicating that the micronutrient homeostasis was maintained, avoiding any negative effects on the PSII. This stability, observed also in **WV**-treated plants, suggests that **WV** did not disrupt **Fe-Mn** balance but rather supported its preservation under increasing stress conditions, consistent with a controlled *eustress* response. The **K/Ca** reduction observed in **NS** (**WV_1_**) and **MS** conditions (**WV_1_** and **WV_2_**) suggests that the **WV** favored **Ca** accumulation, indicating a controlled physiological reorganization typical of a *eustress* response.

This pattern shows that **WV** helps the plants to regulate their cation balance as long as the stress remains within an acceptable range. Contrary, at **HS**, the absence of variation in **WV**-treated plants indicates that the **WV** was no longer able to regulate the stress, pointing to the onset of *distress.* This evidence was supported by the observed results in gas exchange parameters: together with the reduction in **A** and **g_sw_**, **Ci** increased in **HS,** highlighting a phenomenon typical of the onset of *distress* [32,58]. Moreover, the stability of chlorophyll is coherent with this general overview, since in the early stages of the *distress*, functional alterations are mainly observed before structural changes, such as pigment degradation [59,60,61,62].

## 3. Materials and Methods

### 3.1. Wood Vinegar Characteristics

The **WV** used in this study was provided by Bio-Esperia S.r.l. (Arezzo, Italy) [63]. It was obtained through the pyrolysis (650 °C) of five different woody materials (*Castanea sativa* Mill., *Robinia pseudoacacia* L., *Fraxinus* L., *Alnus glutinosa* (L.) Gaertn., and *Quercus robur* L.). The chemical characteristics of this **WV** are reported in Table 3.

### 3.2. Experimental Design

The experiment was conducted from April to July 2025 in a climate-controlled chamber (23 ± 1 °C; 70 ± 5% RH; 350 mmol m^−2^ s^−1^ PAR; 16/8 h day/night) on strawberry plants (*Fragaria × ananassa,* cv ANIA) at the vegetative stage, purchased from a local nursery (Castiglion Fiorentino, Arezzo, Tuscany, Italy). The cultivar used does not present documented drought- or disease-resistance traits and was therefore selected as a representative commercial genotype to evaluate physiological responses to water deficit and biostimulant application. The plants (*n* = 72) were grown individually in plastic pots (10 × 10 × 10 cm) filled with commercial growing medium (Vigor Plant Srl, Italy; the physicochemical characteristics are reported in Celletti et al. [65]). Once in the laboratory, the plants were subjected to three different WV treatments: (*i*) plants grown without the addition of WV (UTC, control plants); (*ii*) plants fertigated with WV at 0.5% (*v*/*v*) (WV_1_); and (*iii*) plants foliar sprayed with WV at 0.2% (*v*/*v*) (WV_2_).

Each treatment was further divided into three water-deficit stress levels applying different water holding capacity (WHC): (*i*) no stress (NS, plants grown with 70% WHC); (*ii*) moderate stress (MS, plants grown with 50% WHC); (*iii*) high stress (HS, plants grown with 30% WHC).

Soil moisture was kept at the target water-deficit stress level for the entire experiment period (3 months) by measuring the weight of each pot daily and adding water only as needed to reach the corresponding WHC for each treatment. WV was applied once a week for a total of twelve applications.

### 3.3. Gas Exchange Parameters

Before the harvest of the plants, leaf gas exchange parameters were measured on fully expanded leaves using a LI-6800 Portable Photosynthesis System (Li-Cor 6800; Li-Cor Inc., Lincoln, NE, USA [66]). For each treatment, eight plants were evaluated, with one measurement taken per plant (*n* = 72). All measurements were conducted between 11:00 and 14:00. Inside the leaf chamber, CO_2_ was maintained at 400 μmol mol^−1^, and airflow was set to 600 μmol s^−1^. Data acquisition was performed once steady-state gas exchange was reached, after CO_2_ and H_2_O fluxes became constant. Data on net CO_2_ assimilation rate (A; expressed as µmol m^−2^ s^−1^), intercellular CO_2_ (Ci; expressed as µmol mol^−1^), stomatal conductance (g_sw_; expressed as mol m^−2^ s^−1^), and transpiration rate (E; expressed as µmol m^− 2^ s^− 1^) were collected. Based on the value acquired and according to Rico et al. [67], the intrinsic water-use efficiency (WUE), expressed as the ratio between A/g_sw_ (µmol CO_2_ mol^−1^ H_2_O), was calculated.

### 3.4. Chlorophyll Content

The total chlorophyll content (Chl tot; expressed as mg m^−2^) was measured on fully expanded leaves using a portable, non-invasive chlorophyll content meter (CCM-300, Opti-Sciences Inc., Hudson, NH, USA [68]). For each leaf, three readings were taken, avoiding the main leaf nerves [64].

### 3.5. Nutrient Balance Ratios

The content of potassium (K), calcium (Ca), iron (Fe), and manganese (Mn) was quantified using a portable X-ray fluorescence analyzer (Olympus, Waltham, MA, USA [69,70,71]). At the end of the experiment, the leaves were oven dried (70 °C for 56 h; Perez-Harguindeguy et al. [72]) and ground with a professional mixer (IKA A10, IKA‑Werke GmbH & Co. KG, Staufen im Breisgau, Germany) to obtain a pulverized material. Subsequently, ca. 1 g of powder was inserted in the instrument compartment for the determination of the elements. The accuracy of the measurements was estimated following the calibration reported in Fedeli et al. [73]. The K/Ca and Fe/Mn nutrient balance ratios were calculated as indicators of the plant’s capacity to preserve ionic homeostasis and discriminate between *eustress* and *distress* conditions.

### 3.6. Statistical Analysis

A permutational analysis of variance (PERMANOVA) was performed to assess the effect of wood vinegar and water stress. When the PERMANOVA indicated a significant (*p* < 0.05) result, a pairwise permutation *t*-test (*p* < 0.05) was applied for post hoc comparisons. RStudio was used to run all the statistical analyses [74]. A heatmap was generated to visualize the multivariate response patterns across treatments relative to their respective controls. Data handling and preprocessing were performed in RStudio version 2026.01.0+392 using the tidyverse package suite. The heatmap was generated with the *pheatmap* and *RColorBrewer* packages, which allowed standardized scaling of variables and the application of a continuous red–yellow–green color gradient reflecting decreasing to increasing values relative to the control (NS). The data matrix was row-centered to improve comparability among traits, and clustering was disabled in order to preserve the experimental treatment order. All other figures were generated using *ggplot2*, with graphical enhancements supported by the *scales*, *cowplot*, and *ggpubr* packages to improve readability, consistency.

## 4. Conclusions and Future Directions

This study showed that wood vinegar (WV), applied via fertigation or foliar spray, elicits a coherent suite of physiological and biochemical adjustments in strawberry plants, consistent with a eustress-driven mechanism of action. Under no-stress and moderate water-deficit conditions, WV reduced gas exchange while increasing WUE, without affecting chlorophyll content or micronutrient homeostasis, indicating a controlled optimization of water use rather than photosynthetic impairment. Nutrient balance ratios further confirmed regulated ionic adjustments characteristic of beneficial low-level stress. Only under high water deficit did WV lose its regulatory capacity, showing early signs of distress, although without structural damage to the photosynthetic apparatus.

These results should be interpreted considering this study’s limitations, including the use of a single species and cultivar and the focus on short-term physiological indicators. Accordingly, the proposed mechanism is inferred rather than directly demonstrated.

Future studies integrating multi-cultivar trials, long-term agronomic outcomes, and molecular or hormonal analyses are needed to further validate the eustress hypothesis.

## Figures and Tables

**Table 1:** Results of PERMANOVA analysis on transpiration rate (**E**), net CO_2_ assimilation rate (**A**), intercellular CO_2_ concentration (**Ci**), stomatal conductance (**g_sw_**), intrinsic water-use efficiency (**WUE**), total chlorophyll content (**Chl tot**), and nutrient balance ratios (**Fe/Mn** and **K/Ca**). Water–deficit stress levels (**WS**) and wood vinegar (**WV**). *df*: degree of freedom; SS: sum of square; R^2^: coefficient of determination; and *F-value*: ratio of explained to unexplained variance. * = *p* < 0.05; ** = *p* < 0.01; *** = *p* < 0.001.

			E			A	
	*df*	SS	R^2^	*F-value*	SS	R^2^	*F-value*
**WS**	2	0.65	0.08	4.96 **	0.29	0.05	2.61
**WV**	2	2.21	0.28	17.0 ***	1.67	0.29	15.1 ***
**WS × WV**	4	0.93	0.12	3.56 **	0.28	0.05	1.29
**Residual**	63	4.11	0.52		3.48	0.61	
**Total**	71	7.9	1.00		5.73	1.00	
			**Ci**			**g_sw_**	
	*df*	SS	R^2^	*F-value*	SS	R^2^	*F-value*
**WS**	2	0.04	0.13	6.95 **	0.72	0.08	4.91 **
**WV**	2	0.02	0.05	2.77	2.34	0.27	16.1 ***
**WS × WV**	4	0.08	0.25	6.96 ***	1.12	0.13	3.85 **
**Residual**	63	0.19	0.57		4.60	0.52	
**Total**	71	0.33	1.00		8.78	1.00	
			**WUE**			**Chl tot**	
	*df*	SS	R^2^	*F-value*	SS	R^2^	*F-value*
**WS**	2	0.31	0.1	5.60 **	0.03	0.11	4.82 *
**WV**	2	0.31	0.1	5.53 **	0.02	0.08	3.41 *
**WS × WV**	4	0.62	0.21	5.50 ***	0.02	0.06	1.25
**Residual**	63	1.76	0.59		0.22	0.75	
**Total**	71	3.00	1.00		0.29	1.00	
		**Fe/Mn**			**K/Ca**		
	*df*	SS	R^2^	*F-value*	SS	R^2^	*F-value*
**WS**	2	0.08	0.09	3.52 *	0.10	0.12	5.14 **
**WV**	2	0.10	0.10	4.15 *	0.02	0.02	0.97
**WS × WV**	4	0.05	0.05	1.11	0.09	0.11	2.34
**Residual**	63	0.73	0.76		0.63	0.74	
**Total**	71	0.96	1.00		0.85	1.00	

**Table 2:** Nutrient balance ratios (**Fe/Mn**–**K/Ca**) expressed as mean ± standard error. **UTC** = plants untreated; **WV_1_** = plants fertigated with wood vinegar at 0.5% (*v*/*v*); **WV_2_** = plants foliar sprayed with wood vinegar at 0.2% (*v*/*v*). **NS** = no drought stress, **MS** = mild drought stress, and **HS** = high drought stress. Different letters indicate statistically significant (*p* < 0.05) differences between treatments.

Treatment	Water Stress Level	Fe/Mn	K/Ca
**UTC**	**NS**	0.34 ± 0.03 ^ab^	1.01 ± 0.09 ^abc^
	**MS**	0.34 ± 0.02 ^a^	1.08 ± 0.04 ^a^
	**HS**	0.26 ± 0.02 ^c^	0.77 ± 0.07 ^c^
**WV_1_**	**NS**	0.27 ± 0.03 ^abc^	0.84 ± 0.04 ^bc^
	**MS**	0.29 ± 0.02 ^abc^	0.91 ± 0.05 ^bc^
	**HS**	0.27 ± 0.02 ^bc^	0.85 ± 0.06 ^bc^
**WV_2_**	**NS**	0.36 ± 0.03 ^a^	1.09 ± 0.12 ^ab^
	**MS**	0.34 ± 0.03 ^ab^	0.89 ± 0.06 ^bc^
	**HS**	0.30 ± 0.01 ^abc^	0.82 ± 0.03 ^c^

**Table 3:** Main chemical characteristics of the wood distillate (Fedeli et al. [64]).

Parameter	Value	Method
TOC (% DW)	58.03	CHNS Elemental Analysis
TN (% DW)	1.06	CHNS Elemental Analysis
H (% DW)	7.27	CHNS Elemental Analysis
S (% DW)	0.07	CHNS Elemental Analysis
pH	4.00	UNI EN ISO 10523:2012
Density (g mL^−1^)	1.05	
Flash point (°C)	>60	ASTM D6450-16°
Total organic compounds (g L^−1^)	33.8	
Acidity (mg L^−1^)	1289.0	APAT CNR IRSA 2010 B Man 29 2003
Organic acids (mg L^−1^)	32.3	
Acetic acid (mg L^−1^)	21.5	
Polyphenols (g L^−1^)	24.5	
Phenols (g L^−1^)	3.00	
PCBs (mg L^−1^)	<0.2	CNR IRSA 24b Q 64 Vol 3 1988
Hydrocarbons C < 12 (mg L^−1^)	<0.1	EPA 5021A 2014 + EPA 8015D 2003
Hydrocarbons C10–C40 (mg L^−1^)	<0.1	UNI EN ISO 9377-2:2002
**16 US-EPA PAHs (mg L^−1^)**		EPA 3550C 2007 + EPA 8310 1986
Acenaphthene	<0.05	
Acenaphthylene	<0.05	
Anthracene	<0.05	
Benzo[a]anthracene	<0.05	
Benzo[a]pyrene	<0.05	
Benzo[b]fluoranthene	<0.05	
Benzo[g,h,i]perylene	<0.05	
Benzo[k]fluoranthene	<0.05	
Chrysene	<0.05	
Dibenz[a,h]anthracene	<0.05	
Fluoranthene	<0.05	
Fluorene	<0.05	
Indeno[1,2,3-cd]pyrene	<0.05	
Naphthalene	<0.05	
Phenanthrene	<0.05	
Pyrene	<0.05	
**Macronutrients (mg L ^−1^)**		Alkaline melting + ICP-MS analysis
Ca	325.50	
K	23.49	
Mg	6.79	
P	7.28	
**Micronutrients (mg L ^−1^)**		Alkaline melting + ICP-MS analysis
Cu	0.18	
Fe	21.16	
Mn	0.58	
Mo	0.0007	
Zn	3.22	
**Other nutrients**		Alkaline melting + ICP-MS analysis
Al	1.96	
Ba	0.06	
Cr	0.03	
Na	103.59	

**TOC**: total organic carbon. **TN**: total nitrogen. **PCBs**: polychlorinated biphenyls. **16 US-EPA PAHs**: list of 16 priority polycyclic aromatic hydrocarbons as classified by the United States Environmental Protection Agency. **Al**: aluminum; **Ba**: barium; **C**: carbon; **Ca**: calcium; **Cr**: chromium; **Cu**: copper; **Fe**: iron; **K**: potassium; **Mg**: magnesium; **Mn**: manganese; **Mo**: molybdenum; **N**: nitrogen; **Na**: sodium; and **Zn**: zinc.

## Data Availability

Data are available upon reasonable request from the corresponding author. The data are not publicly available due to privacy and ethical restrictions.

## References

[1] Grewal A., Abbey L., Gunupuru L.R. (2018). Production, prospects and potential application of pyroligneous acid in agriculture. J. Anal. Appl. Pyrolysis.

[2] Aguirre J.L., Baena J., Martín M.T., Nozal L., González S., Manjón J.L., Peinado M. (2020). Composition, ageing and herbicidal properties of wood vinegar obtained through fast biomass pyrolysis. Energies.

[3] Iacomino G., Idbella M., Staropoli A., Nanni B., Bertoli T., Vinale F., Bonanomi G. (2024). Exploring the potential of wood vinegar: Chemical composition and biological effects on crops and pests. Agronomy.

[4] Wei Q., Ma X., Dong J. (2010). Preparation, chemical constituents and antimicrobial activity of pyroligneous acids from walnut tree branches. J. Anal. Appl. Pyrolysis.

[5] Zhu K., Gu S., Liu J., Luo T., Khan Z., Zhang K., Hu L. (2021). Wood vinegar as a complex growth regulator for rapeseed. Agronomy.

[6] Maresca V., Fedeli R., Vannini A., Munzi S., Corrêa A., Cruz C., Loppi S. (2024). Wood distillate enhances seed germination. Appl. Sci..

[7] Mungkunkamchao T., Kesmala T., Pimratch S., Toomsan B., Jothityangkoon D. (2013). Wood vinegar and fermented bioextracts enhance growth and yield of tomato. Sci. Hortic..

[8] Benzon H.R.L., Lee S.C. (2016). Potential of wood vinegar in enhancing fruit yield and antioxidant capacity in tomato. Korean J. Plant Resour..

[9] Fedeli R., Dichiara M., Carullo G., Tudino V., Gemma S., Butini S., Campiani G., Loppi S. (2024). Unlocking the potential of biostimulants in sustainable agriculture: Effect of wood distillate on the nutritional profiling of apples. Heliyon.

[10] Ofoe R., Mousavi S.M.N., Thomas R.H., Abbey L. (2024). Foliar application of pyroligneous acid synergizes with fertilizer in tomato. Sci. Rep..

[11] European Commission European Green Deal. https://commission.europa.eu/strategy-and-policy/priorities-2019-2024/european-green-deal_en.

[12] European Commission Farm to Fork. https://food.ec.europa.eu/horizontal-topics/farm-fork-strategy_en.

[13] Scopus Data. https://www.scopus.com/.

[14] Luo X., Wang Z., Meki K., Wang X., Liu B., Zheng H., You X., Li F. (2019). Effect of co-application of wood vinegar and biochar on seed germination and seedling growth. J. Soils Sediments.

[15] Mirsoleimani A., Najafi-Ghiri M., Boostani H.R., Heydari H. (2024). Effect of wood vinegar on vegetative growth and nutrient uptake in two citrus rootstocks. J. Hortic. Postharvest Res..

[16] De Guzman R.S., Dadural M.I.Y. (2021). Seed germination and seedling growth of mango as affected by different concentrations of wood vinegar. Galaxy Int. Interdiscip. Res. J..

[17] Theapparat Y., Chandumpai A., Faroongsarng D. (2018). Physicochemistry and utilization of wood vinegar from tropical biomass. Tropical Forests.

[18] Fedeli R., Cruz C., Loppi S., Munzi S. (2024). Hormetic effect of wood distillate on hydroponically grown lettuce. Plants.

[19] Fedeli R., Loppi S., Cruz C., Munzi S. (2024). Evaluating seawater and wood distillate for sustainable hydroponic cultivation: Implications for crop growth and nutritional quality. Sustainability.

[20] Fedeli R., Marotta L., Frattaruolo L., Panti A., Carullo G., Fusi F., Saponara S., Gemma S., Butini S., Cappello A.R. (2023). Nutritionally enriched tomatoes (*Solanum lycopersicum* L.) grown with wood distillate: Chemical and biological characterization. J. Food Sci..

[21] Bienertova-Vasku J., Lenart P., Scheringer M. (2020). Eustress and distress: Neither good nor bad, but rather the same?. BioEssays.

[22] Kupriyanov R., Zhdanov R. (2014). The eustress concept: Problems and outlooks. World J. Med. Sci..

[23] Lichtenthaler H.K. (1998). The stress concept in plants: An introduction. Ann. N. Y. Acad. Sci..

[24] Zhuang D., Li H.B., Wang Y., Zhou D., Zhao L. (2025). Nanoparticle-elicited eustress intensifies cucumber adaptation to water deficit. Environ. Sci. Technol..

[25] Caicedo-Lopez L.H., Guevara-Gonzalez R.G., Ramirez-Jimenez A.K., Feregrino-Perez A.A., Contreras-Medina L.M. (2022). Eustress application through elicitation for capsaicinoids enhancement: A review. Phytochem. Rev..

[26] El-Nakhel C., Pannico A., Kyriacou M.C., Giordano M., De Pascale S., Rouphael Y. (2019). Macronutrient deprivation eustress elicits secondary metabolites in lettuce. J. Sci. Food Agric..

[27] Noel R., Schueller M.J., Ferrieri R.A. (2024). Radiocarbon flux measurements reveal why pyroligneous acid stimulates plant growth. Int. J. Mol. Sci..

[28] He Z., Zhang P., Jia H., Zhang S., Nishawy E., Sun X., Dai M. (2024). Regulatory mechanisms and breeding strategies for crop drought resistance. New Crops.

[29] Franco-Navarro J.D., Padilla Y.G., Álvarez S., Calatayud Á., Colmenero-Flores J.M., Gómez-Bellot M.J., Hernández J.A., Martínez-Alcalá I., Penella C., Pérez-Pérez J.G. (2025). Advancements in water-saving strategies and crop adaptation to drought: A comprehensive review. Physiol. Plant..

[30] Chaves M.M., Oliveira M.M. (2004). Mechanisms underlying plant resilience to water deficits: Prospects for water-saving agriculture. J. Exp. Bot..

[31] Baker N.R. (2008). Chlorophyll fluorescence: A probe of photosynthesis in vivo. Annu. Rev. Plant Biol..

[32] Flexas J., Medrano H. (2002). Drought-inhibition of photosynthesis in C3 plants: Stomatal and non-stomatal limitations revisited. Ann. Bot..

[33] Chaves M.M., Flexas J., Pinheiro C. (2009). Photosynthesis under drought and salt stress: Regulation mechanisms from whole plant to cell. Ann. Bot..

[34] Ma Y., Yuan Z., Wei Z., Yan F., Liu X., Li X., Hou J., Hao Z., Liu F. (2025). Stomatal and non-stomatal regulations of photosynthesis in response to salinity, and K and Ca fertigation in cotton (*Gossypium hirsutum* L cv.). Environ. Exp. Bot..

[35] Salmon Y., Lintunen A., Dayet A., Chan T., Dewar R., Vesala T., Hölttä T. (2020). Leaf carbon and water status control photosynthetic limitations. New Phytol..

[36] Hur G., Ashraf M., Nadeem M.Y., Rehman R.S., Thwin H.M., Shakoor K., Seleiman M.F., Alotaibi M., Yuan B.-Z. (2025). Exogenous application of wood vinegar improves rice yield and quality by elevating photosynthetic efficiency. Plant Physiol. Biochem..

[37] Mohd Amnan M.A., Teo W.F.A., Aizat W.M., Khaidizar F.D., Tan B.C. (2023). Foliar application of oil palm wood vinegar enhances *Pandanus amaryllifolius* tolerance under drought stress. Plants.

[38] Abdel-Sattar M., Mostafa L.Y., Rihan H.Z. (2024). Enhancing mango productivity with wood vinegar, humic acid, and seaweed extract applications as an environmentally friendly strategy. Sustainability.

[39] Afsharipour S., Seyedi A., Dastjerdi A.M. (2025). Salinity tolerance in *Cucumis sativus* seedlings: The role of pistachio wood vinegar on the improvement of biochemical parameters and seedlings vigor. BMC Plant Biol..

[40] Rapacz M., Wójcik-Jagła M., Fiust A., Kalaji H.M., Kościelniak J. (2019). Genome-wide associations of chlorophyll fluorescence OJIP parameters in barley. Front. Plant Sci..

[41] Oxborough K., Baker N.R. (1997). Resolving chlorophyll a fluorescence images of photosynthetic efficiency. Photosynth. Res..

[42] Lootens P., Van Waes J., Carlier L. (2004). Effect of a short photoinhibition stress on photosynthesis. Photosynthetica.

[43] Zhou H., Fu K., Shen Y., Li R., Su Y., Deng Y., Xia Y., Zhang N. (2024). Wood vinegar-induced stress response against tomato Fusarium wilt. Plants.

[44] Ye Y., Sun H., Wang Y., Xu Z., Han S., He G., Yin K., Zhang H. (2022). Wood vinegar alleviates photosynthetic inhibition under *Pseudomonas syringae* infection. J. Plant Interact..

[45] Zhang T., Li Y., Tang Y., Ding Y., Rui Y. (2025). Mechanisms of wood vinegar alleviating salt stress in wheat. Agronomy.

[46] Junlin Z., Guangjie Z., Yi T., Wenli L., Chang Y., Yanan X., Deshun X., Guang C., Chumney X., Danying W. (2025). Wood vinegar enhances the survival rate of rice seeds under flooding stress. Rice Sci..

[47] Fedeli R., Vannini A., Grattacaso M., Loppi S. (2023). Wood distillate (pyroligneous acid) boosts nutritional traits of potato tubers. Ann. Appl. Biol..

[48] Nestby R., Lieten F., Pivot D., Lacroix C.R., Tagliavini M. (2005). Influence of mineral nutrients on strawberry fruit quality. Int. J. Fruit Sci..

[49] Kabata-Pendias A. (2000). Trace Elements in Soils and Plants.

[50] Khan M.I.R., Nazir F., Maheshwari C., Chopra P., Chhillar H., Sreenivasulu N. (2023). Mineral nutrients in plants under changing environments. Plant Genome.

[51] Gerretsen F.C. (1949). Manganese in relation to photosynthesis: I carbon dioxide assimilation and manganese deficiency symptoms of oats. Plant Soil..

[52] Somers I.I., Shive J.W. (1942). The iron–manganese relation in plant metabolism. Plant Physiol..

[53] Marschner H. (2011). Marschner’s Mineral Nutrition of Higher Plants.

[54] Mengel K., Kirkby E.A. (2012). Principles of Plant Nutrition.

[55] Osmolovskaya N., Shumilina J., Bureiko K., Chantseva V., Bilova T., Kuchaeva L., Laman N., Wessjohann L.A., Frolov A. (2019). Ion homeostasis response to nutrient-deficiency stress in plants. Cell Growth.

[56] Qu R., Han G. (2022). Effects of high Ca and Mg stress on plant water-use efficiency. PeerJ.

[57] Xu C., Yang H., Huang C., Lan M., Zou Z., Zhang F., Zhang L. (2023). Interaction mechanism of Fe, Mg and Mn in karst soil–mango system. Land.

[58] Farquhar G.D., Sharkey T.D. (1982). Stomatal conductance and photosynthesis. Annu. Rev. Plant Physiol..

[59] Akley E.K., Ampim P.A., Obeng E., Sanyare S., Yevu M., Owusu Danquah E., Amoako O.A., Tengey T.K., Avedzi J.K., Avornyo V.K. (2023). Wood vinegar promotes soil health and the productivity of cowpea. Agronomy.

[60] Krasensky J., Jonak C. (2012). Drought, salt, and temperature stress-induced metabolic rearrangements. J. Exp. Bot..

[61] Mittler R. (2006). Abiotic stress, the field environment and stress combination. Trends Plant Sci..

[62] Villagómez-Aranda A.L., Feregrino-Pérez A.A., García-Ortega L.F., González-Chavira M.M., Torres-Pacheco I., Guevara-González R.G. (2022). Activating stress memory: Eustressors in plant breeding. Plant Cell Rep..

[63] (2025). BioDea. https://biodea.bio/prodotto/distillato-di-legno-bio/.

[64] Fedeli R., Celletti S., Loppi S. (2024). Wood distillate promotes the tolerance of lettuce in extreme salt stress conditions. Plants.

[65] Celletti S., Fedeli R., Ghorbani M., Aseka J.M., Loppi S. (2023). Exploring sustainable alternatives: Wood distillate alleviates the impact of bioplastic in basil plants. Sci. Total Environ..

[66] Licor 2025; LI-6800 Portable Photosynthesis System Instruction Manual. Version 3, 2019. https://www.licor.com/products/photosynthesis/LI-6800.

[67] Rico E.I., de la Fuente G.C.M., Morillas A.O., Ocaña A.M.F. (2024). Drought tolerance in 14 olive cultivars. Photosynth. Res..

[68] Opti-Sciences CCM-300 Chlorophyll Content Meter. Opti-Sciences Inc.: Hudson, NH, USA, 2025. https://opti-sciences.com/products/content-meters/ccm-300/.

[69] Olympus. Olympus Corporation. (s.d.) X-Ray Fluorescence Analyzer. Olympus Corporation: Waltham, MA, USA, 2025. https://www.olympus-europa.com/company/country-home/.

[70] Et-Tazy L., Fedeli R., Khibech O., Lamiri A., Challioui A., Loppi S. (2025). Effects of monoterpene-based biostimulants on chickpea (*Cicer arietinum* L.) plants: Functional and molecular insights. Biology.

[71] Fedeli R., Zhatkanbayeva Z., Loppi S. (2025). Wood distillate as a solution for growing crops under water deficiency. Crops.

[72] Perez-Harguindeguy N., Diaz S., Garnier E., Lavorel S., Poorter H., Jaureguiberry P., Cornelissen J.H.C. (2016). Corrigendum: New handbook for standardised measurement of plant functional traits worldwide. Aust. J. Bot..

[73] Fedeli R., Di Lella L.A., Loppi S. (2024). Suitability of XRF for routine analysis of multi-elemental composition: A multi-standard verification. Methods Protoc..

[74] R Core Team (2025). R: A Language and Environment for Statistical Computing.

